# Alumni Meetings

**Published:** 1893-01

**Authors:** 


### Alumni Meetings.

The gathering of the profession at Chicago, next August, will afford an unusual opportunity for the Alumni of the different colleges of the country to come together, to hold meetings, to renew acquaintance, and to discuss and talk over matters of interest to each other.

Those colleges whose Alumni have an organization will find this an easy matter to accomplish, and those who have not yet formed Alumni Associations should call the graduates of the respective colleges for such meetings at that time.

The question with the Alumni of any given college would be, who shall take the initiative in calling them together? We would here suggest that two or three of the more active members of the classes of 1892 could very properly take this work in hand, as they would feel quite as much, if not more, interest in it than the older graduates, and they could probably have easier access to the entire list of graduates of their respective colleges. It is very desirable that work for such meetings be taken up at once. It would be a very interesting occasion for not only the Alumni, but for the profession generally; it would also add interest to the great occasion.

We would be glad, in any way possible, to give aid to such a series of meetings.

